# Pericentromeric recombination suppression and the ‘large X effect’ in plants

**DOI:** 10.1038/s41598-023-48870-3

**Published:** 2023-12-07

**Authors:** Edgar L. Y. Wong, Dmitry A. Filatov

**Affiliations:** 1 Department of Biology, University of Oxford, Oxford, UK; 2 Senckenberg Biodiversity and Climate Research Centre, Frankfurt am Main, Germany

**Keywords:** Evolutionary genetics, Speciation

## Abstract

X chromosome was reported to be a major contributor to isolation between closely related species—the ‘large X’ effect (LXE). The causes of LXE are not clear, but the leading theory is that it is caused by recessive species incompatibilities exposed in the phenotype due to the hemizygosity of X-linked genes in the heterogametic sex. However, the LXE was also reported in species with relatively recently evolved sex chromosomes where Y chromosome is not completely degenerate and X-linked genes are not hemizygous, such as the plant *Silene latifolia*. Recent genome sequencing and detailed genetic mapping in this species revealed a massive (> 330 Mb) non- or rarely-recombining pericentromeric region on the X chromosome (Xpr) that comprises ~ 90% of the chromosome and over 13% of the entire genome. If any of the Xpr genes are involved in species incompatibilities, this would oppose interspecific gene flow for other genes tightly linked in the Xpr. Here we test the hypothesis that the previously reported LXE in *S. latifolia* is caused by the lack of recombination on most of the X chromosome. Based on genome-wide analysis of DNA polymorphism and gene expression in *S. latifolia* and its close cross-compatible relative *S. dioica*, we report that the rarely-recombining regions represent a significant barrier for interspecific gene flow. We found little evidence for any additional factors contributing to the LXE, suggesting that extensive pericentromeric recombination suppression on the X-chromosome is the major if not the only cause of the LXE in *S. latifolia* and *S. dioica*.

## Introduction

Hybridisation and gene flow between closely related species is common and evolution of reproductive barriers is the crucial step in speciation process. In animals, sex chromosomes are known to play a disproportionately large role in isolation between incipient species (e.g.,^1–3^). Interspecific hybridisation often leads to asymmetric outcome, with hybrid inviability and sterility usually occurring in the heterogametic sex—the observation that is often called *Haldane’s rule* (HR), indicating that sex chromosomes play a major role in speciation^4,5^. The X-chromosome was also proposed to have disproportionately large role in dysfunction of hybrids in comparison to their autosomal counterparts, known as the *large X effect* (LXE)^6–8^. The LXE and HR are often referred to as the “two rules of speciation”.

The LXE and HR, are thought to be caused by recessive species incompatibilities exposed in the phenotype due to the hemizygosity of X-linked genes in the heterogametic sex^7,9^. Thus, the reports of HR and the LXE in species with recently evolved non or partially-degenerate Y-chromosomes^10^, such as *Silene latifolia* and its relatives discussed below^11,12^, were surprising and cast doubts that hemizygous X-linked genes are the universal major cause of HR and LXE. Other possible causes of HR and LXE include meiotic drive on sex chromosomes^13–15^, misregulation of X-chromosome in hybrids^1,3^, quicker evolution of genes linked to X-chromosome (*faster-X theory*; e.g.,^16–18^), higher density of male sterility loci on X chromosomes than autosomes^1^, quicker evolution of spermatogenesis-related genes and stronger sexual selection exerted on males than females (*faster males theory*;^19^).

Here we analyse another possible cause of the LXE—the presence of a massive block of rarely- or non-recombining DNA on the X-chromosome, as recently reported for *S. latifolia*^20^. Extensive pericentromeric recombination suppression (PRS) on very large (~ 400Mb) *S. latifolia* X-chromosome appears to be an extreme case of a general tendency for long chromosomes to have large central chromosomal regions with rare recombination. The reasons for this are not clear, but they are discussed in the literature^21,22^. Regions of low recombination often show high genetic differentiation between species because stronger and wider linkage disequilibrium (LD) in such regions increases linkage of loci involved in interspecific incompatibility (barrier loci) with a larger chunk of the genome, which leads to suppressed introgression in such regions (e.g.,^23–29^). Non-recombining regions could contribute to the maintenance of species integrity despite on-going interspecific hybridisation, as noted in many theoretical and empirical studies (e.g.,^23,30–34^). Some suggested that suppression of introgression in low-recombining regions is key to maintaining divergence between hybridising species (e.g.,^35,36^).

The focal species of this study, *S. latifolia* and *S. dioica*, are commonly found across Europe. Habitat differentiation plays a crucial role in reproductive isolation between the two species^37^: *S. latifolia* inhabiting open fields and road margins, while *S. dioica* is more common in shady and moist habitats. They also differ in a number of phenotypic traits, including but not limited to flower colour, size and shape of sepal and seed capsules, and leaf shape^38^. Although the two species form viable and fertile hybrids where they co-occur^39,40^, some fitness reduction in hybrids (such as low pollen viability) had been detected^11^. *S. latifolia* and *S. dioica* have indistinguishable karyotypes, with the Y being the largest and the X the second largest chromosome in the genome^41,42^. The separate sexes and sex chromosomes are of relatively recent origin in this lineage—they have evolved about ~ 11 million years ago in the ancestor of *S. latifolia* and *S. dioica*, as estimated from synonymous divergence between the X- and Y-linked gametologs based on the mutation rate that was measured directly in *S. latifolia*^43^. Although some degeneration was reported on the Y chromosome^43–47^, most sex-linked genes are not hemizygous in males. This raises the question how the ‘two rules of speciation’, reported for these species^11,12^, apply to species with such recently evolved sex chromosomes. One possibility is that rapid species-specific degeneration of Y-linked genes and associated adjustment of expression of X-linked gametologs (dosage compensation) may lead to rapid evolution of sex-linked species incompatibilities^44^. This model is particularly suitable for species with large, recently evolved sex chromosomes, such as in *S. latifolia* and *S. dioica*, because the rate of Y-degeneration is proportional to the number of genes linked together in a non-recombining region^48^, so it has to be fast for young sex chromosomes and slow down once only few functional Y-linked genes are left, as inferred for mammalian Y chromosomes (Fig. 4 in^49^).

Recent sequencing of *S. latifolia* genome and its integration with high-density genetic map^20^ revealed substantial pericentromeric recombination suppression (PRS) on all chromosomes. PRS is particularly extensive on the X chromosome, where the rarely-recombining pericentromeric region (Xpr) comprises at least 330 Mb, which is ~ 90% of the X chromosome length and over 13% of the total genome length. Recombination rates are similar in male and female meiosis in *S. latifolia*^50^ and extensive PRS is unrelated to heterochiasmy, but PRS may have contributed to evolution of recombination suppression between the nascent X- and Y-chromosomes in this species^51^. As explained above, the rarely- or non-recombining regions represent a significant obstacle in interspecific gene flow. If most of the X chromosome in *S. latifolia* (and likely in *S. dioica*) is represented by a massive rarely-recombining block of chromatin impenetrable to interspecific gene flow, this may be the main reason for the LXE reported for these species^12^. Here we test this hypothesis to evaluate whether the presence of the massive rarely-recombining region in the *S. latifolia* X chromosome is sufficient to explain the LXE. Specifically, we compared patterns of polymorphisms and gene expression divergence between rarely-recombining X-linked genes and other X-linked and autosomal genes. We also employed demographic modelling to characterise the extent of gene flow in different parts of the *Silene* genome.

## Materials and methods

### Transcriptome dataset

The analyses in this study are based on sequence data from 12 *S. latifolia* and 12 *S. dioica* females (Table 1) grown in the glasshouse (20 °C, 15-h lighting) from seeds collected in the wild. Actively growing shoots with flower buds were used for total RNA extraction with a Qiagen RNeasy Plant Mini Kit with on-column DNase digestion. Isolation of mRNA, cDNA synthesis and high-throughput sequencing were conducted according to the standard Illumina RNA-Seq procedure at the WTCHG genomics facility (Oxford, UK). The resulting sequence reads were mapped to female reference transcriptome^46^ that was also used in the genetic mapping^50,52^. Read mapping was done with BWA mem 0.7.17^53^ and sorted with Samtools 1.7^54^. Then, SNP calling was done with Samtools mpileup (options: -d 1000 -q 20 -Q 20) and sites filtered with bcftools filter 1.7. The resulting multisample vcf file was converted to fasta alignments using ProSeq software^55^ available from https://sourceforge.net/projects/proseq/. The latter software was also used for further processing and analysis of resulting datasets. Gene expression was quantified as per-gene FPKM (fragments per kilobase per million reads), calculated with RSEM^56^.

**Table 1:** *Silene* samples used in this study. Newly submitted samples are part of the BioProejct PRJNA1012686.

Name	Species	Country	Region	Accession	References
benF	*S. latifolia*	Austria	near Klagenfurt	SRS994834	^52^
f833d	*S. latifolia*	Spain	Broto	SRS242366	^46^
ERR4643711	*S. latifolia*			ERR4643711	^70^
ERR4643713	*S. latifolia*			ERR4643713	^70^
fSa1179	*S. latifolia*	UK	Oxfordshire	SAMN37270384	This study
fSa1180	*S. latifolia*	UK	Oxfordshire	SAMN37270385	This study
fSa1181	*S. latifolia*	UK	near Oxford	SAMN37270386	This study
fSa1182	*S. latifolia*	UK	Oxfordshire	SAMN37270387	This study
fSa2056	*S. latifolia*	UK	near Oxford	SAMN37270388	This study
fSa615	*S. latifolia*	Germany	near Leipzig	SAMN37270389	This study
fSa331a	*S. latifolia*	UK	North England	SAMN37270390	This study
fSa1596	*S. latifolia*	UK	Oxfordshire	SAMN37270391	This study
fSd1167	*S. dioica*	UK	Brill	SAMN37270392	This study
fSd1170	*S. dioica*	UK	near Brill	SAMN37270393	This study
fSd1171	*S. dioica*	UK	Oxfordshire	SAMN37270394	This study
fSd1175	*S. dioica*	UK	near Brill	SAMN37270395	This study
fSd1176	*S. dioica*	UK	Oxford	SAMN37270396	This study
fSd1177	*S. dioica*	UK	Oxfordshire	SAMN37270397	This study
fSd2047	*S. dioica*	UK	near Brill	SAMN37270398	This study
fSd2081	*S. dioica*	UK	Scottish border	SAMN37270399	This study
fSd33b	*S. dioica*	UK	Wales	SAMN37270400	This study
fSd468C	*S. dioica*	Austria	St Oswald	SAMN37270401	^12^
fSd496a	*S. dioica*	Austria	road to Mariazel	SAMN37270402	^12^
fSd554d	*S. dioica*	Austria	Trounkirchen	SAMN37270403	This study

The following groups of genes were used in the analyses: rarely-recombining autosomal genes (rareA); rarely-recombining X-linked genes in the Xpr region (rareX); frequently-recombining autosomal genes (freqA); frequently-recombining X-linked genes in the qXdr region (freqX). These groups were defined according to the location of a gene in the rarely recombining central chromosome region or actively recombining ends of the chromosomes, based on the *S. latifolia* female genome sequence^20^. Pericentromeric recombination suppression is extensive on all *S. latifolia* chromosomes^20,50^ and genetic analysis detected no recombination in the central regions of the chromosomes, while recombination at the ends of the chromosomes was frequent^50^. As the transition between the frequently recombining ends of the chromosomes and rarely-recombining central regions is quite sharp^20^, we reasoned the split of the genes in the freqA, rareA, freqX and rareX categories is well justified.

### Genomewide polymorphism statistics and comparisons between gene categories

Five polymorphism indices, namely nucleotide diversity (*π*)^57^, *Tajima’s D*^58^, *F*_*ST*_^57^, *D*_*xy*_^57^ and *Z*_*nS*_^59^, were measured using ProSeq^55^ for all sites, fourfold degenerate sites and the first two codon positions. The fourfold degenerate sites are considered the most neutral type of sites in the genome (e.g. Fig. 2 in reference^60^), while the first two codon positions are likely least neutral. All the above statistics were firstly plotted against genomic positions (using the R package *ggplot2*;^61^) to obtain a genome wide overview (fourfold degenerate *π* and *Tajima’s D*; all sites for *F*_*ST*_, *D*_*xy*_ and *Z*_*nS*_). Then, their values were compared based on the following categories using the Kruskal–Wallis test and Wilcoxon rank-sum test: (1) between frequently-recombining and rarely-recombining groups of genes analysed separately within each species (for *Tajima’s D* and *π*; using fourfold degenerate sites, or first two codon positions; and for *Z*_*nS*_ using all sites) or between the two species (for *D*_*xy*_ and *F*_*ST*_; using all sites); (2) among frequently-recombining and rarely-recombining genes in autosomes and X chromosome (from the frequently-recombining qXdr region and rarely-recombining Xpr region), respectively. These statistics were also estimated for each chromosome using all sites, fourfold degenerate sites, and first two codon positions, respectively. To correct for ploidy difference in comparisons between the X-linked and the autosomal genes the estimates of *π* in autosomes were adjusted to 75% of the original values. Both adjusted and original values are reported here and whenever this correction is used, it is explicitly stated in the text.

### Demographic modelling

**Figure 1:**
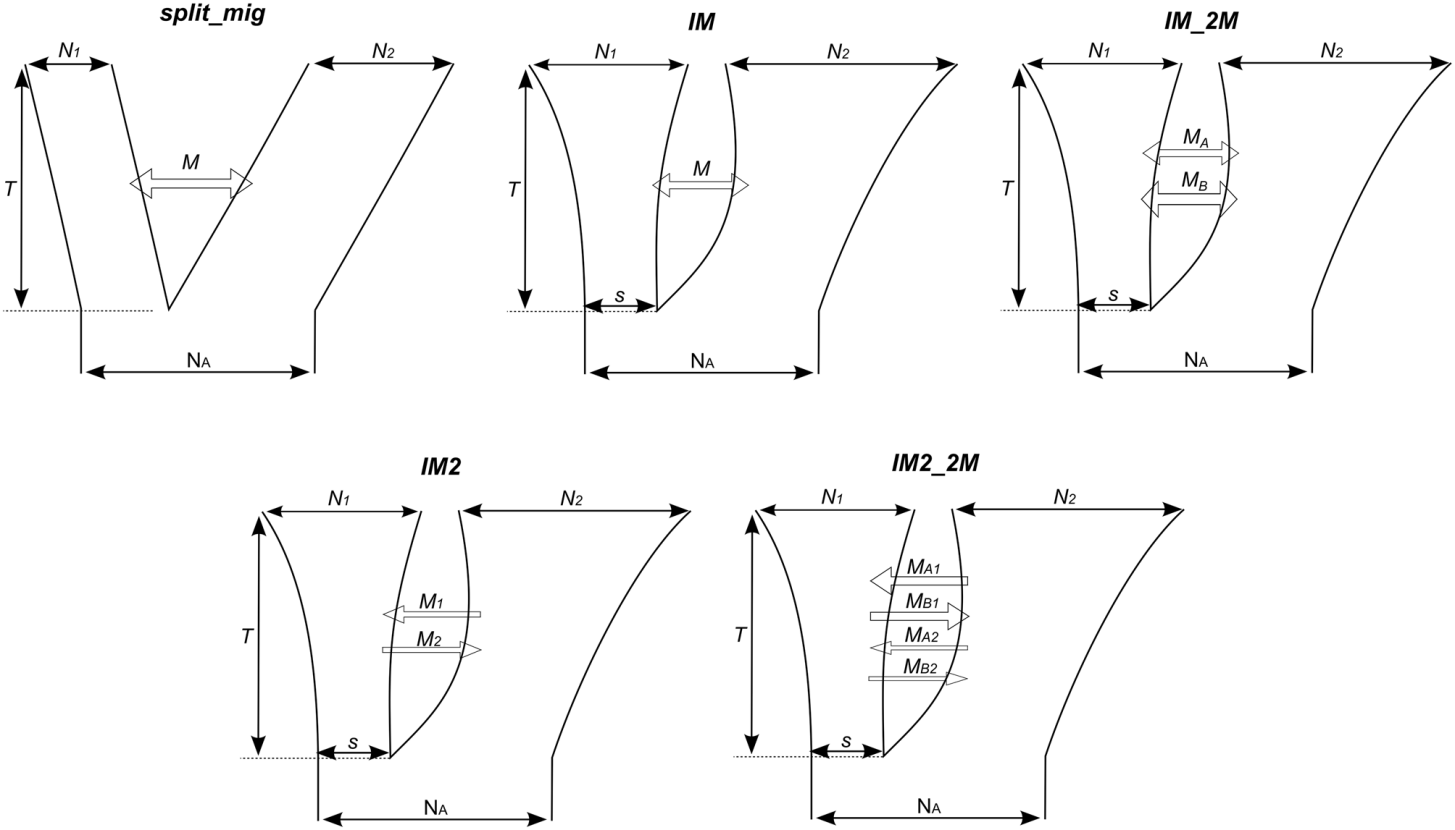
Schematic representation of the five demographic models used in this study. In each diagram, the width of the tree branches at the top shows the current population sizes (N_1_ and N_2_), and moving down (backward in time) the inferred demographic history since the species split. The model that assumes constant population size (split_mig) is represented by straight lines. Models that allow for exponential population size changes since the split (IM, IM_2M, IM2, IM2_2M) have curved lines and include the parameter *s*, which is the relative size of the population 1 at the split (relative size of population 2 is 1-s). N_A_ is the ancestral population size before the split, and is not a free parameter^63^. All population sizes (N_1_ and N_2_) are expressed in units of N_A_. The time parameter, T, is given in units of 2*N_A_ generations. All migration parameters (M, M_1_, M_2_, M_A_, M_B_, M_A1_, M_A2_, M_B1_, M_B2_) are represented by horizontal arrows and expressed in units of 2*N_A_*m, where m is the proportion of the receiving population consisting of immigrants in each generation. The “A” and “B” indexes for migration parameters reflect migration rate at two classes of sites in the genome in the IM_2M and IM2_2M models.

To quantify and compare the extent of gene flow in rarely- and frequently-recombining genes in the two *Silene* species, we used five demographic models (from^62^) that utilise Poisson random field-based demography inference framework implemented in dadi package^63^. These models include (Fig. 1): split_mig—population split with bi-directional migration and constant population size; IM—population split (isolation) with bi-directional migration equal in two directions and population size change; IM_2M—IM with bi-directional heterogeneous migration that is allowed to differ between two classes of sites across the genome; IM2—IM with migration allowed to differ in two directions; IM2_2M—IM2 with heterogeneous migration for two classes of sites (Fig. 1). The models with heterogeneous migration (IM_2M and IM2_2M) include two categories of genomic sites with different migration parameters. These models were chosen to test whether the two species had experienced significant population size change since divergence, whether gene flow differed in each direction and whether there was heterogeneous gene flow (presence of this would potentially mean significant differences in gene flow between autosomes and the X chromosome in each recombination category). Heterogeneous gene flow was tested using two sets of nested models – IM versus IM_2M, and IM2 versus IM2_2M. The fit of these models to data was compared with likelihood ratio tests (LRT). All these models were run for frequently-recombining and rarely-recombining genes separately. Additionally, models IM2 and IM2_2M were run for the following groups of genes: rarely-recombining autosomal genes (rareA); rarely-recombining X-linked genes in the Xpr region (rareX); frequently-recombining autosomal genes (freqA); frequently-recombining X-linked genes in the qXdr region (freqX). Ten initial runs were performed for each model with a wide parameter range (0–5 for time parameters, 0–10 for migration parameters, 0–100 for population size parameters). Based on estimated parameter values in these initial runs, parameter ranges were adjusted for a further 30 runs. The best-fitting model (the run with the highest estimated likelihood) was selected based on Akaike Information Criterion (AIC). Robustness of parameter estimates of the best-fitting models was evaluated with 100 bootstrap runs, with the confidence intervals calculated as M ± 1.96X (where M is the likelihood parameter estimate and X is the standard deviation of parameter estimates from the bootstrap runs).

## Results

### Significant differences in polymorphism statistics between frequently and rarely-recombining genes

**Figure 2:**
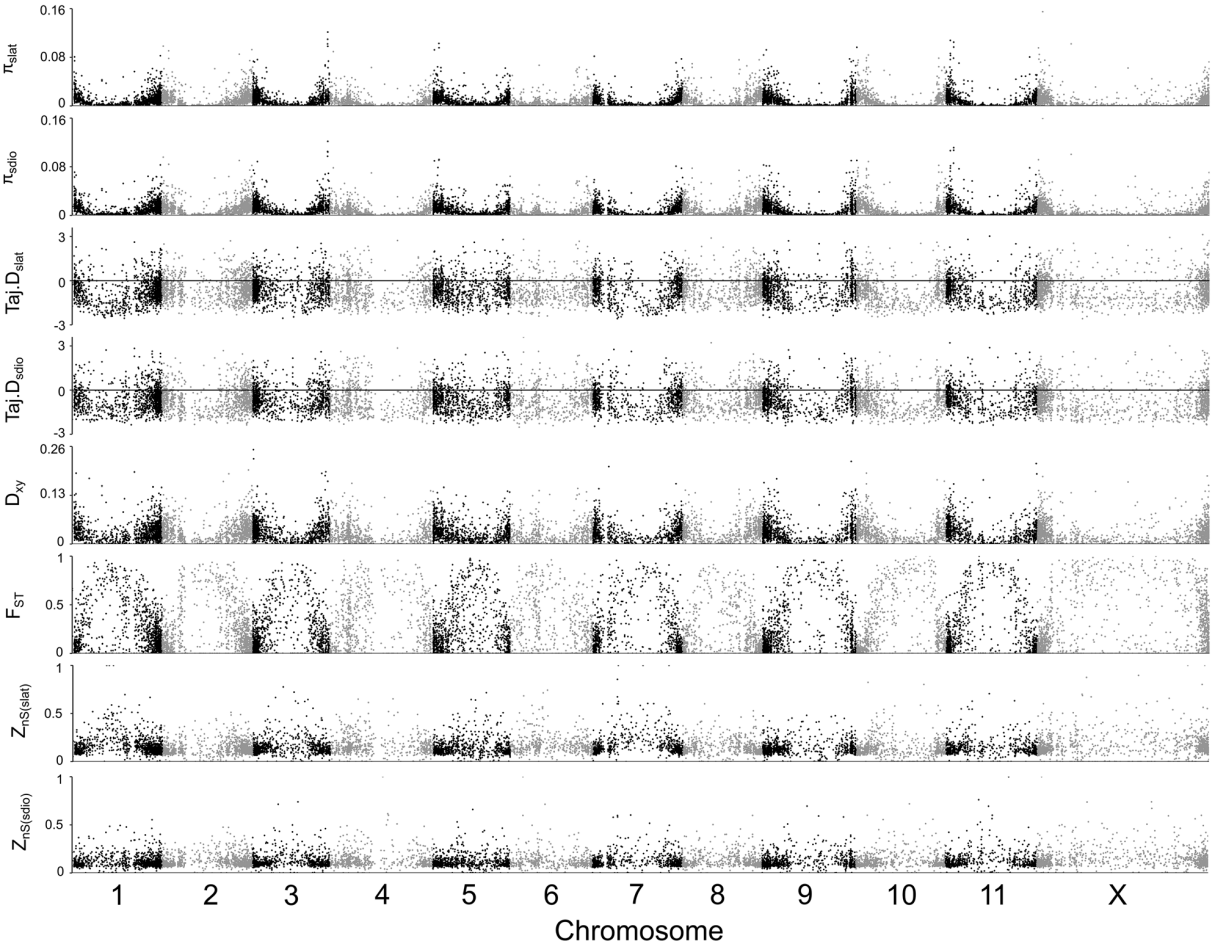
Genomewide polymorphism statistics in *S. latifolia* (slat) and *S. dioica* (sdio). From top to bottom panel: nucleotide diversity (*π*) of each species (fourfold degenerate sites), *Tajima’s D* for each species (fourfold degenerate sites), *D*_*xy*_ and *F*_*ST*_ between the two species (all sites) and *Z*_*nS*_ for each species (all sites).

The distribution of genetic diversity was similar in *S. latifolia* and *S. dioica* genomes (Spearman’s correlation for *π* (fourfold degenerate sites): R = 0.99, *p*-value < 2.2 × 10^–16^; Spearman’s correlation for *π* (first two codon positions): R = 0.99, *p*-value < 2.2 × 10^–16^; Spearman’s correlation for *Tajima’s D* (fourfold degenerate sites): R = 0.30, *p*-value < 2.2 × 10^–16^; Spearman’s correlation for *Tajima’s D* (first two codon positions): R = 0.22, *p*-value < 2.2 × 10^–16^). Genetic diversity varied considerably across both genomes, with the highest diversity observed at the ends of the chromosomes and much lower diversity in the central regions (Fig. 2). This corresponds to the distribution of recombination rate reported for *S. latifolia* genome, with extensive pericentromeric recombination suppression present on all chromosomes and frequent recombination occurring only near the ends of all chromosomes^20,50^. Consistent with this, the extent of linkage disequilibrium, quantified with *Z*_*nS*_ statistic^59^, was higher in the central regions of the chromosomes compared to actively recombining ends of the chromosomes (Fig. 2).

Below we analyse and compare the patterns of DNA polymorphism separately for frequently and rarely-recombining regions. The genes lying in the central chromosomal regions, where no recombination was detected in genetic cross data^20,50^ are designated as “rarely” or “low”-recombining, while the genes located in the actively recombining ends of the chromosomes are designated as “frequently” or “high”-recombining, with similar numbers of genes analysed in these categories (2161 and 2261, respectively). At fourfold degenerate sites, median *π* was 0.0324 and 0.0341 in the frequently recombining autosomal genes (freqA); and 0.0107 and 0.0118 in the rarely recombining autosomal genes (rareA) in *S. latifolia* and *S. dioica*, respectively (Supp. Table 2). Median *π* at fourfold degenerate sites on the X chromosome was 0.0227 and 0.0209 in the frequently recombing genes (freqX); 0.0029 and 0.0048 in the rarely recombining genes (rareX) in *S. latifolia* and *S. dioica*, respectively (Supp. Table 2). In the first two codon positions, median *π* was 0.0053 in the frequently recombining genes for both species; and 0.0023 and 0.0026 in the rarely recombining genes in *S. latifolia* and *S. dioica*, respectively (Supp. Table 3). Median *π* in the first two codon positions in freqX genes were 0.0043 and 0.0039; and 0.0011 and 0.0016 in rareX genes of the two species (Supp. Table 3). Median *F*_*ST*_ (all sites) was the highest in both freqX and rareX genes (Supp. Table 1). Median *D*_*xy*_ (all sites) was the second lowest and lowest in freqX and rareX genes, respectively (Supp. Table 1). Median *Z*_*nS*_ of freqX genes was highest for both species, but that of rareX genes was highest only for *S. dioica* (Supp. Table 1).

**Figure 3:**
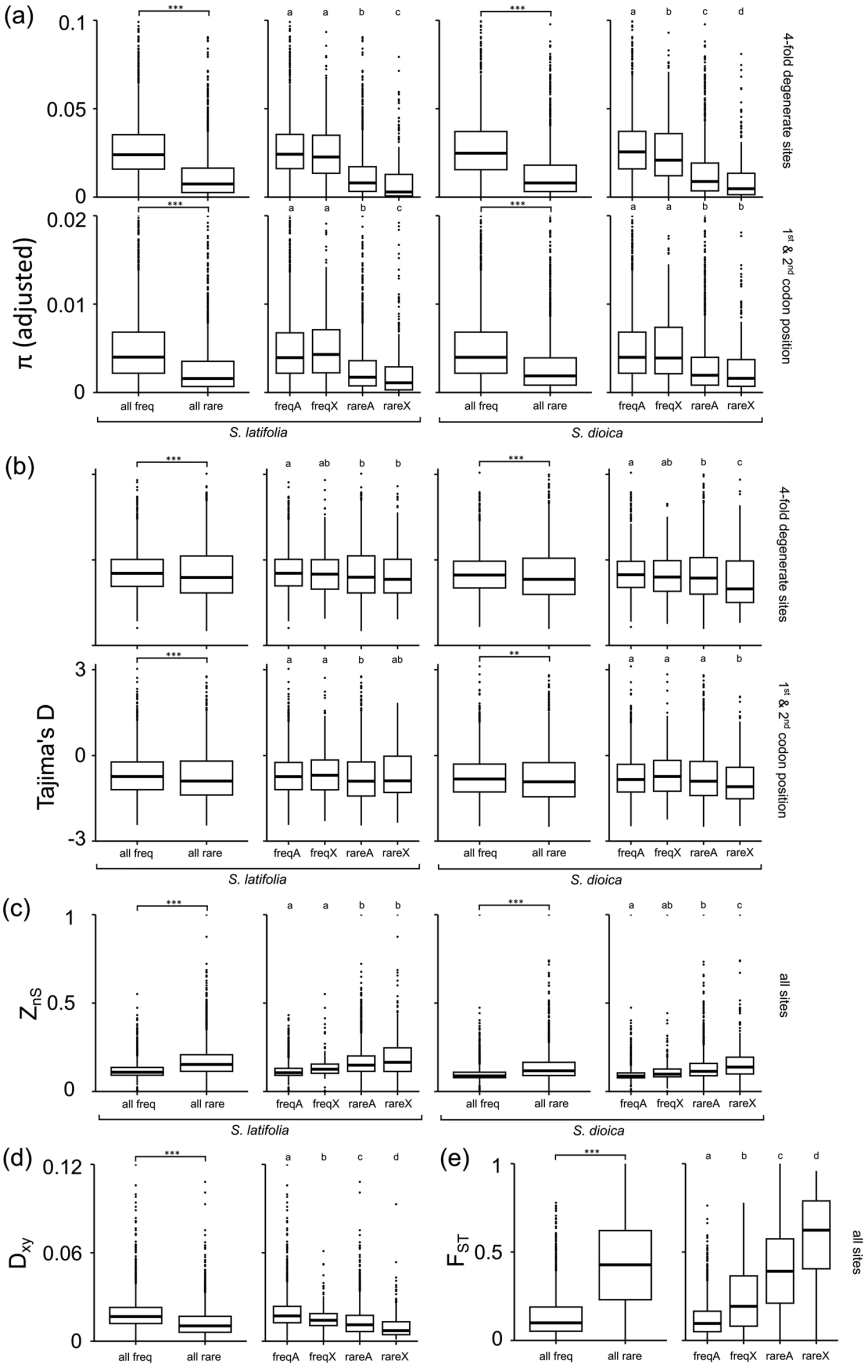
Comparisons of polymorphism statistics between different groups of autosomal and X-linked genes in the frequently- and rarely-recombining genomic regions of the two *Silene* species. Letters (**a**–**d**) at the top of each box plot represent groupings based on the Wilcoxon rank-sum test. In the Kruskal–Wallis test, *indicates *p*-value < 0.05, **indicates *p*-value < 0.005, *** indicates *p*-value < 0.0005. Top of plots for *π* were cut off for better resolution of differences among groups. Full *π* plots and plots with adjusted *π* (0.75 of estimated values for autosomal genes) are presented in Supp. Figs. 1 and 2, respectively.

The frequently and rarely-recombining regions differed in the level and patterns of DNA polymorphism, with *π* (fourfold degenerate sites and first two codon positions), *Tajima’s D* (fourfold degenerate sites and first two codon positions), *D*_*xy*_ (all sites), *F*_*ST*_ (all sites), *Z*_*nS*_ (all sites) all showed significant differences between these regions (Fig. 3). *π*, *D*_*xy*_ and *F*_*ST*_ were also significantly different for all pairwise comparisons in autosomal and X-linked genes in the two recombination categories (Fig. 3, Supp. Fig. 2). However, after autosomal genes’ *π* had been adjusted for ploidy difference with X (by multiplying each value by 0.75), the same pattern remained significant only for fourfold degenerate sites in *S. dioica* (Fig. 3a, Supp. Fig. 1). RareX genes had significantly different adjusted *π* from all other groups in fourfold degenerate sites for both species, and first two codon positions in *S. latifolia* (Fig. 3a, Supp. Fig. 1). In the first two codon positions in *S. dioica*, rareX genes had significantly different adjusted *π* from freqA and freqX, but not rareA genes (Fig. 3a, Supp. Fig. 1). For *Tajima's D*, rareX genes did not differ significantly from rareA and freqX genes in *S. latifolia*; whereas rareX genes in *S. dioica* differ significantly from all three other groups (freqA, rareA and freqX) (Fig. 3b). In *S. latifolia*, *Z*_*nS*_ differed significantly between freq (A or X) and rare (A or X) genes but not within recombination categories (between A and X). In *S. dioica*, the patterns are similar to that of *Tajima’s D* that rareX genes differed significantly with all other groups (Fig. 3b).

### Demographic modelling

In order to exclude the effect of pericentromeric recombination suppression on gene flow we conducted separate analyses for rarely-recombing and frequently-recombining regions. For each of these datasets we fitted two pairs of nested models, IM and IM_2M, IM2 and IM2_2M (Fig. 1) that differed in the number of parameters accounting for interspecific gene flow. All these models included population size change after species split, which turns out to be an essential feature of the models, given the model without population size change (split_mig, Fig. 1) showed much lower fit to data compared to any of the models allowing for population size changes (Table 2). The parameter estimates for population size change showed 2.23 to 2.99-fold population size growth in both species (Table 2), which is consistent with north-ward post-glacial expansion of these species from refugia in southern Europe or Anatolia^64^.

**Table 2:** Best parameter estimates of demographic models analysed with dadi, Akaike information criterion (AIC), and results of the likelihood ratio tests (LRT) for nested models (IM versus IM_2M and IM2 versus IM2_2M). The parameters are as in Fig. 1. Confidence levels for parameters of the best-fitting model for each gene category are shown (± 1.96X, where X = standard deviation in 100 bootstrap estimates).

Gene category	Model	No. of free param	log-like	θ	AIC	ΔAIC	Rel. like	(LRT) 2ΔLL	(LRT) *p*-value	s	N_1_		
Rarely-recombining genes (rare)	split_mig	4	− 9717	23147	19441	15841	0.00				0.69		
	IM	5	− 8249	18385	16507	12907	0.00			0.26	1.34		
	IM_2M	7	− 2710	17227	5433	1833	0.00	11,078	0.00	0.26	1.38		
	IM2	6	− 8235	18259	16481	12882	0.00			0.43	1.33		
	**IM2_2M**	***9***	***− 1791***	***9461***	***3600***	***0***	***1.00***	***12,888***	***0.00***	***0.62* ± *0.41***	***2.29* ± *1.12***		
Frequently-recombining genes (freq)	split_mig	4	− 5686	37420	11380	8117	0.00				1.13		
	IM	5	− 4434	37078	8878	5615	0.00			0.70	1.58		
	IM_2M	7	− 1789	34987	3592	329	0.00	5290	0.00	0.60	1.59		
	IM2	6	− 4431	40,578	8875	5611	0.00			0.13	1.75		
	**IM2_2M**	***9***	***− 1623***	***20533***	***3263***	***0***	***1.00***	***5617***	***0.00***	***0.28* ± *0.37***	***2.23* ± *0.99***		
Rarely-recombining, autosomal genes (rareA)	IM2	6	− 5576	13,081	11164	8116	0.00			0.43	1.22		
	**IM2_2M**	***9***	***− 1515***	***7470***	***3048***	***0***	***1.00***	***8122***	***0.00***	***0.27* ± *0.21***	***1.82* ± *0.80***		
Rarely-recombining, X-linked genes in Xpr region (rareX)	IM2	6	− 1649	1803	3311	2129	0.00			0.72	0.77		
	**IM2_2M**	***9***	***− 582***	***1109***	***1182***	***0***	***1.00***	***2135***	***0.00***	***0.29* ± *0.21***	***1.20* ± *0.62***		
Frequently-recombining, autosomal genes (freqA)	IM2	6	− 3687	29,379	7386	4723	0.00			0.35	3.78		
	**IM2_2M**	***9***	***− 1323***	***25831***	***2664***	***0***	***1.00***	***4729***	***0.00***	***0.38* ± *0.37***	***1.38* ± *0.92***		
Frequently-recombining, X-linked genes in qXdr region (freqX)	IM2	6	− 1306	3879	2623	1445	0.00			0.82	1.20		
	**IM2_2M**	***9***	***− 580***	***2477***	***1179***	***0***	***1.00***	***1451***	***0.00***	***0.65* ± *0.43***	***1.62* ± *1.03***		

Gene category	Model	N_2_	T	M	M_1_	M_2_	M_A_	M_B_	M_A1_	M_A2_	M_B1_	M_B2_	P
rare	split_mig	0.85	1.32	0.46									
	IM	1.29	1.96	0.32									
	IM_2M	1.41	2.37				0.12	1.71					0.55
	IM2	1.16	2.14		0.24	0.43							
	**IM2_2M**	***2.99* ± *1.03***	***6.17* ± *2.15***						***0.06*** ± 0.03	***0.10*** ± 0.06	***2.12*** ± 1.30	***1.21*** ± 0.65	***0.56*** ± 0.22
freq	split_mig	1.28	0.54	1.34									
	IM	2.41	0.63	1.16									
	IM_2M	2.01	0.84				0.23	2.22					0.19
	IM2	2.03	0.45		2.56	0.62							
	**IM2_2M**	***2.78* ± *0.75***	***3.43* ± *1.85***						***0.25*** ± 0.13	***0.18*** ± 0.08	***2.03*** ± 1.06	***1.39*** ± 0.61	***0.26*** ± 0.07
rareA	IM2	1.18	1.58		0.32	0.41							
	**IM2_2M**	***2.48* ± *0.85***	***4.84* ± *1.53***						***0.10*** ± 0.04	***0.11*** ± 0.05	***2.52*** ± 1.16	***0.75*** ± 0.70	***0.55*** ± 0.19
rareX	IM2	0.59	1.31		0.15	0.74							
	**IM2_2M**	***1.24* ± *0.63***	***3.85* ± *1.92***						***0.04*** ± 0.05	***0.13*** ± 0.09	***3.13*** ± 1.65	***2.88*** ± 1.07	***0.50*** ± 0.21
freqA	IM2	2.17	0.18		0.27	1.22							
	**IM2_2M**	***2.36* ± *1.01***	***0.55* ± *0.31***						***0.38*** ± 0.18	***0.39*** ± 0.34	***7.90*** ± 3.55	***0.52*** ± 0.42	***0.31*** ± 0.26
freqX	IM2	1.57	0.93		0.60	0.86							
	**IM2_2M**	***1.95* ± *0.72***	***3.77* ± *1.79***						***0.15*** ± 0.08	***0.18*** ± 0.09	***2.50*** ± 1.03	***1.26*** ± 0.67	***0.28*** ± 0.14

The best-fitting model and its parameter values for each category are in bold/bolditalics.

IM and IM_2M models assumed that gene flow is the same in both directions, while IM2 and IM2_2M allowed for different migration rates in two directions. Fitting of these models to data revealed that gene flow differs significantly in two directions (Table 2), with *S. latifolia* to *S. dioica* gene flow (M_1_) being stronger than in the opposite direction (M_2_), which is consistent with asymmetric reproductive barrier between these species^65^.

The IM and IM2 models assumed that all sites in the genome had the same gene flow, while the more complex *_2M models allowed for two different classes of sites (“A” and “B”) with different migration rates. Better fit of the *_2M models to data (Table 2) demonstrates the presence of significant heterogeneity in interspecific gene flow across the genome. The “A” sites (M_A_, M_A1_ and M_A2_ in Table 2) show much lower migration rate(s) compared to the "B" sites (M_B_, M_B1_ and M_B2_ in Table 2), with ~ 7 to ~ 70-fold difference between the A and B sites (Table 2). Larger proportion of the analysed sites belonged the lower migration A-category for rarely-recombining regions (56%) compared to frequently recombining regions (26%). This is consistent with rarely-recombining regions representing a significant barrier to interspecific gene flow. Analyses using separate autosomal and X-linked genes from rarely-and frequently recombining regions (“rareA”, “freqA’, “rareX”, “freqA” gene categories), revealed a similar pattern (Table 2)—higher proportions of analysed sites fell into the low migration A-category in the rarely-recombining regions (55% and 50% in “rareA” and “rareX” gene categories, respectively) than in frequently-recombining regions (31% and 28% in “freqA” and “freqX” gene categories, respectively).

The comparison of estimated gene flow for X-linked and autosomal genes reveals that on average (across M_A1_, M_A2_, M_B1_ and M_B2_ in Table 2) for frequently recombining regions, it is about twofold lower on the X compared to autosomes (M_Aut_/M_X_ = 2.1), which is consistent with significantly higher *F*_*ST*_ for freqX compared to freqA (Fig. 3e) as well as with the large-X effect. On the other hand, the rarely-recombining regions show little difference in migration rates between the X-linked and autosomal genes (average M_Aut_/M_X_ = 1.1), indicating that lack of recombination limits gene flow to a similar extent on the X-chromosome and the autosomes. The estimated time since species divergence (measured in generations times twice the ancestral population size) was similar for all categories except the frequently recombining autosomal genes, where it was much lower (T_rareX_ = 3.85; T_freqX_ = 3.77; T_rareA_ = 4.84; T_freqA_ = 0.55, Table 2).

### Gene expression divergence in frequently and rarely-recombining regions

To compare the rate of gene expression divergence on the X chromosome and autosomes, we measured expression in transcriptome sequence data from 12 *S. latifolia* and 12 *S. dioica* females (Table 1). As expected for closely related species, gene expression in the two species was strongly positively correlated (Table 3). The correlation was the strongest for the frequently recombining X-linked genes (r^2^ = 0.870) and the weakest for the rarely-recombining X-linked genes (r^2^ = 0.781), suggesting that gene expression divergence is slightly faster in rarely-compared to frequently recombining X-linked genes (Table 3). However, the proportion of genes that evolved significantly (t-test *P* < 0.0001) different expression was the same (10%) in these categories. This proportion was the lowest (7.22%) in the frequently recombining autosomal genes, while in all other gene categories it was close to 10% (Table 4). Only the difference between frequently and rarely-recombining autosomal genes was marginally significant (chi^2^ = 3.595; *P* = 0.0580) for the number of genes that evolved significantly different expression in the two species. All other pairwise comparisons were non-significant. Taken together, these results indicate that gene expression divergence between *S. latifolia* to *S. dioica* is slowest in the frequently recombining autosomal genes (freqA), possibly due to more active interspecific gene flow homogenising gene pools of these species. Unlike the autosomal frequently recombining genes, expression of the freqX genes is diverging at a similar rate to rarely recombining X-linked genes, which is consistent with the X-linkage acting as a partial barrier to interspecific gene flow.

**Table 3:** Correlation (*r*^2^) of gene expression (FPKM) between *S. latifolia* and *S. dioica*.

	X	A	All
Freq	0.870	0.843	0.836
Rare	0.781	0.789	0.782
All	0.789	0.812	0.805

**Table 4:** The numbers and proportions of genes that evolved significantly (t-test, *P* < 0.0001) different expression between *S. latifolia* and *S. dioica*.

	FreqX^a^	FreqA^b^	RareX^c^	RareA^d^
All	209	2009	398	1909
Diff. expression	21	145	40	172
% Diff expression	10.05%	7.22%	10.05%	9.01%

^a^X-linked genes in the frequently recombining qXdr region.

^b^Autosomal genes in frequently recombining regions.

^c^X-linked genes in the rarely-recombining Xpr region.

^d^Autosomal genes in rarely-recombining regions.

## Discussion

This study analysed the level and patterns of genetic diversity across *S. latifolia* and *S. dioica* genomes to assess the contribution of the extensive pericentromeric recombination suppression to limiting gene flow between these species. Lack of recombination leads to linkage disequilibrium of a barrier locus with a wider genomic region, which leads to suppressed introgression in such regions even for loci that are not causing any hybrid inviability or reduced fertility. Thus, rarely-recombining regions may be major contributors to the maintenance of species integrity despite on-going interspecific hybridisation (e.g.,^23,30–34^). As *S. latifolia* and *S. dioica* regularly hybridise and introgress in overlapping ranges across Europe, rarely-recombining regions, especially that on the X-chromosome, could be key to maintaining their distinct species identities.

We conducted analyses of genetic diversity, interspecific divergence and gene flow separately for regions with 'high' and 'low' recombination rates. While this division of the genome into two classes may appear crude, it does reflect strong differences in recombination rate at the ends and central regions of all chromosomes. Pericentromeric recombination suppression is quite extensive on all *S. latifolia* chromosomes, with the central rarely-recombining region comprising most of the length of all chromosomes^20,50^. This division into a very large (~ 330 Mb) rarely-recombining central region and small frequently recombining regions at the ends is particularly pronounced on the X-chromosome, which is the largest in the female genome^20^. The transition between the frequently recombining ends of the chromosomes and rarely-recombining central regions is quite sharp^20^, and a few genes falling in the transition zones with intermediate recombination rate were excluded from our analysis. Given this distribution of recombination across the *S. latifolia* genome, the artificial division into 'high' and 'low' (or 'freq' and 'rare') recombination classes reflects biological reality well.

Genetic diversity was observed to be substantially lower in the rarely-recombining central regions of all chromosomes, compared to actively recombining chromosomal ends. Reduced diversity in rarely-recombining regions is a general phenomenon likely caused by linked selection—selective sweeps^66^ and background selection^67^ that affect wider genomic regions in rarely-recombining regions due to stronger linkage disequilibrium. Selective sweeps are expected to drive allele frequency spectrum towards the excess of low frequency polymorphisms, which is detectable by negative Tajima's D values^68^. This statistic is indeed more negative in rarely-recombining compared to frequently recombining regions (Fig. 3b; Supp. Tables 1, 2, 3). In particular, rareX genes had significantly more negative *Tajima’s D* than both freqA and freqX genes in *S. dioica* (both fourfold degenerate sites and first two codon positions)*,* and freqA genes in *S. latifolia* (fourfold degenerate sites only) (Fig. 3b, Supp. Table 2, 3). Genetic diversity in the X-linked genes is lower compared to the autosomes, as expected from their ploidy difference. The lower ploidy for X-linked genes accounts for their lower diversity in frequently recombining X-linked regions (*π* for freqX and freqA genes were similar after adjustment for the difference in ploidy, except for fourfold degenerate sites in *S. dioica*), but it is not sufficient to explain reduced diversity in the massive rarely-recombining Xpr region on the X chromosome, compared to rarely-recombining autosomal regions. Even after adjusting *π* for autosomal genes, rareX genes still had significantly lower *π* than rareA genes in both species and types of analysed sites, except for those in *S. dioica* from first two codon positions (Fig. 3a, Supp. Tables 2, 3).This may be due to particularly large size of the Xpr (~ 330Mb) that includes over thousand genes^20^, which should make linked selection that reduces genetic diversity particularly strong. Indeed, linkage disequilibrium (measured with *Z*_*nS*_) is strongest in rareX genes (Supp. Table 1).

Genetic differentiation between *S. latifolia* and *S. dioica*, measured with *F*_*ST*_, is higher in the rarely-recombining central regions of the chromosomes, compared to actively recombining terminal regions with rareX genes having significantly higher *F*_*ST*_ than all other groups (Fig. 3e; Supp. Table 1). This is likely caused by reduced gene flow, but the reduced intraspecific genetic diversity in rarely-recombining regions (Figs. 2, 3, Supp. Tables 1, 2, 3) could have also contributed to higher *F*_*ST*_ by increasing the relative proportion of overall genetic diversity that is due to species divergence. Lower *D*_*xy*_ in the central compared to peripheral regions of the chromosomes (Figs. 2, 3, Supp. Table 1) is also indicative that high *F*_*ST*_ in the central rarely-recombining regions is, at least partly, caused by reduced intraspecific genetic diversity. However, the demographic modelling reveals consistently lower estimates of interspecific gene flow in the rarely-recombining compared to frequently recombining regions (Table 2). Time since species divergence estimated for rarely- and frequently recombining X-linked genes is very similar (T_rareX_ = 3.85; T_freqX_ = 3.77; Table 2), which indicates similar coalescent times for the two groups of X-linked genes and suggests that rare recombination and X-linkage both act as considerable interspecific barriers. This is also consistent with much lower T for frequently—(T_freqA_ = 0.55) compared to rarely—(T_rareA_ = 4.84) recombining autosomal regions, with higher interspecific gene flow in the former compared to the latter (Table 2), homogenising gene pools of the two species and reducing T.

While rarely-recombining regions appear to represent significant barriers to interspecific gene flow, X-linkage may also contribute significantly to species differentiation as indicated by higher *F*_*ST*_ values in the X-linked compared to autosomal genes for regions with similar recombination rates (i.e. rareX versus rareA, and freqX versus freqA; Fig. 3e). However, *D*_*xy*_ is lower in the X-linked compared to autosomal genes both for frequently- and rarely-recombining regions (Fig. 3d), indicating that higher *F*_*ST*_ for X-linked genes is at least partly caused by lower intraspecific genetic diversity on the X-chromosome. Furthermore, the fitting of demographic models to data did not show significantly lower gene flow for X-linked compared to autosomal genes for regions with similar recombination rate (Table 2). The proportion of sites (*P*) falling into low gene flow category was similar for X-linked and autosomal genes within the same recombination category (*P*_rareX_ = 0.50 vs *P*_rareA_ = 0.55; *P*_freqX_ = 0.28 vs *P*_freqA_ = 0.31; Table 2). Thus, the effect of X-linkage (if any) on gene flow appears to be much less pronounced compared to reduced recombination rate in pericentromeric regions.

## Conclusion

In this study, we tested whether the pericentromeric recombination suppression in the massive Xpr region^20^ on the *S. latifolia* X chromosome can account for the LXE previously reported for this species^12^. While LXE in animals has been shown with direct experiments^69^, the evidence for LXE in *S. latifolia*^12^ and our analyses presented above are indirect—based on evolutionary genetic analyses of genetic diversity and gene flow between the species. We report that population differentiation (*F*_*ST*_; Fig. 3e) and the proportion of sites with low interspecific gene flow (*P* in Table 2) are significantly higher in the rarely-recombining compared to the actively recombining regions on the X-chromosome and the autosomes. This reveals an important role of the rarely-recombining regions in limiting gene flow between the two species. As the rarely-recombining region comprises a larger proportion of the X-chromosome (~ 90%) compared to the autosomes (~ 80%)^20^, this likely disproportionately reduces overall interspecific gene flow on the X, contributing to the 'large-X' effect. We found little evidence that X-linkage by itself contributes significantly to the LXE in *S. latifolia* and *S. dioica*. The frequently recombining part of the X-chromosome does have a significantly higher *F*_*ST*_ compared to the frequently recombining regions on the autosomes (Fig. 3e), but this appears to be caused by lower genetic diversity in the X-linked genes. We conclude that the lack of recombination in pericentromeric regions creates a significant barrier for interspecific gene flow, which is a cause for the LXE in *S. latifolia* and *S. dioica* due to a disproportionately large pericentromeric region on the X-chromosome.

### Supplementary Information

Supplementary Information.

## Data Availability

Previously unpublished sequences were uploaded to GenBank under the BioProejct PRJNA1012686.
