# Supplementary material for: Pericentromeric recombination suppression and the ‘large X effect’ in plants
Source: Sci Rep. 2023 Dec 7;13:21682. doi: 10.1038/s41598-023-48870-3 (PMC10709461; doi:10.1038/s41598-023-48870-3)
Supplement: Supplementary file 1 — Supplementary Information. [file 41598_2023_48870_MOESM1_ESM.docx]

**Supplementary Tables and Figures**

**Pericentromeric recombination suppression and the 'large X effect' in plants**

**Edgar L.Y. Wong^1,2^ and Dmitry A. Filatov^1^**

^1^Department of Biology, University of Oxford, Oxford, UK

^2^Senckenberg Biodiversity and Climate Research Centre, Frankfurt am Main, Germany

Correspondence: [dmitry.filatov@biology.ox.ac.uk](mailto:dmitry.filatov@biology.ox.ac.uk)

Supp Table 1. Summary of polymorphism statistics (median) for all sites in each chromosome.

| **Frequently-recombining genes** | | | | | | | | | | | | | | |
| --- | --- | --- | --- | --- | --- | --- | --- | --- | --- | --- | --- | --- | --- | --- |
| **Chrom.** | **No. of genes** | **Combined length (bp)** | ***S. lat.* π** | ***S. lat.* π - SD** | ***S. dio.* π** | ***S. dio.* π - SD** | ***S. lat.* adj. π** | ***S. dio.* adj. π** | ***S. lat.* Taj. D** | ***S. dio.* Taj. D** | **F_ST_** | **D_xy_** | ***S. lat.* ZnS** | ***S. dio.* ZnS** |
| **1** | 354 | 230,083 | 0.0137 | 0.0077 | 0.0134 | 0.0082 | 0.0103 | 0.0101 | -0.529 | -0.711 | 0.1185 | 0.0157 | 0.105 | 0.086 |
| **2** | 292 | 185,203 | 0.0139 | 0.0083 | 0.0144 | 0.0082 | 0.0104 | 0.0108 | -0.565 | -0.612 | 0.1180 | 0.0171 | 0.107 | 0.089 |
| **3** | 214 | 140,065 | 0.0165 | 0.0091 | 0.0168 | 0.0097 | 0.0123 | 0.0126 | -0.476 | -0.5885 | 0.1029 | 0.0191 | 0.109 | 0.084 |
| **4** | 24 | 14,748 | 0.0112 | 0.0064 | 0.0124 | 0.0074 | 0.0084 | 0.0093 | -0.501 | -0.291 | 0.1555 | 0.0140 | 0.109 | 0.083 |
| **5** | 56 | 39,095 | 0.0133 | 0.0074 | 0.0162 | 0.0082 | 0.0100 | 0.0121 | -0.57 | -0.429 | 0.0875 | 0.0173 | 0.119 | 0.094 |
| **6** | 16 | 4,128 | 0.0606 | 0.0223 | 0.0566 | 0.0192 | 0.0455 | 0.0425 | 1.333 | 1.759 | 0.0982 | 0.0650 | 0.187 | 0.245 |
| **7** | 214 | 133,140 | 0.0148 | 0.0082 | 0.0150 | 0.0089 | 0.0111 | 0.0113 | -0.412 | -0.562 | 0.0991 | 0.0170 | 0.105 | 0.086 |
| **8** | 176 | 115,054 | 0.0157 | 0.0087 | 0.0155 | 0.0090 | 0.0118 | 0.0116 | -0.397 | -0.467 | 0.0700 | 0.0175 | 0.103 | 0.088 |
| **9** | 201 | 128,497 | 0.0148 | 0.0084 | 0.0155 | 0.0087 | 0.0111 | 0.0116 | -0.5255 | -0.6175 | 0.0775 | 0.0167 | 0.105 | 0.0865 |
| **10** | 117 | 64958 | 0.0153 | 0.0095 | 0.0148 | 0.0094 | 0.0115 | 0.0111 | -0.705 | -0.771 | 0.0399 | 0.0159 | 0.092 | 0.08 |
| **11** | 246 | 141,212 | 0.0155 | 0.0087 | 0.0154 | 0.0090 | 0.0116 | 0.0115 | -0.4965 | -0.5175 | 0.0934 | 0.0173 | 0.108 | 0.089 |
| **all auto.** | 1,910 | 1,196,183 | 0.0148 | 0.0084 | 0.0151 | 0.0089 | 0.0111 | 0.0113 | -0.506 | -0.5795 | 0.0960 | 0.0172 | 0.106 | 0.087 |
| **X** | 351 | 244,297 | 0.0102 | 0.0059 | 0.01 | 0.0058 | 0.0102 | 0.01 | -0.573 | -0.631 | 0.1930 | 0.0142 | 0.126 | 0.098 |
| **Rarely-recombining genes** | | | | | | | | | | | | | | |
| **Chrom.** | **No. of genes** | **Combined length (bp)** | ***S. lat.* π** | ***S. lat.* π - SD** | ***S. dio.* π** | ***S. dio.* π - SD** | ***S. lat.* adj. π** | ***S. dio.* adj. π** | ***S. lat.* Taj. D** | ***S. dio.* Taj. D** | **F_ST_** | **D_xy_** | ***S. lat.* ZnS** | ***S. dio.* ZnS** |
| **1** | 85 | 59,695 | 0.0051 | 0.0037 | 0.0039 | 0.0028 | 0.0038 | 0.0029 | -1.0855 | -0.8778 | 0.4387 | 0.0107 | 0.189 | 0.1015 |
| **2** | 185 | 129,823 | 0.0068 | 0.0042 | 0.0076 | 0.0051 | 0.0051 | 0.0057 | -0.504 | -0.774 | 0.3359 | 0.0128 | 0.1245 | 0.11 |
| **3** | 177 | 121,322 | 0.0064 | 0.0039 | 0.0082 | 0.0048 | 0.0048 | 0.0061 | -0.558 | -0.7085 | 0.3360 | 0.0126 | 0.155 | 0.115 |
| **4** | 164 | 117,803 | 0.0037 | 0.0023 | 0.0037 | 0.0023 | 0.0028 | 0.0028 | -0.8385 | 0.808 | 0.4919 | 0.0076 | 0.1655 | 0.12 |
| **5** | 227 | 159,242 | 0.0054 | 0.0035 | 0.0058 | 0.0035 | 0.0041 | 0.0044 | -0.762 | -0.685 | 0.4051 | 0.0115 | 0.144 | 0.103 |
| **6** | 184 | 133,647 | 0.0058 | 0.0036 | 0.005 | 0.0031 | 0.0044 | 0.0038 | -0.5545 | -0.555 | 0.3798 | 0.0118 | 0.179 | 0.127 |
| **7** | 42 | 37913 | 0.0033 | 0.0025 | 0.0035 | 0.0025 | 0.0025 | 0.0026 | -0.925 | -1.174 | 0.5023 | 0.0074 | 0.194 | 0.1235 |
| **8** | 176 | 131051 | 0.0071 | 0.0045 | 0.0078 | 0.0054 | 0.0053 | 0.0059 | -0.634 | -0.876 | 0.3265 | 0.0126 | 0.1545 | 0.114 |
| **9** | 134 | 103,277 | 0.0064 | 0.0039 | 0.0071 | 0.0043 | 0.0048 | 0.0053 | -0.581 | -0.667 | 0.3367 | 0.0112 | 0.14 | 0.102 |
| **10** | 180 | 139,201 | 0.0031 | 0.0024 | 0.0043 | 0.0031 | 0.0023 | 0.0032 | -1.058 | -1 | 0.4942 | 0.0093 | 0.138 | 0.115 |
| **11** | 235 | 148,499 | 0.0061 | 0.0037 | 0.0065 | 0.0041 | 0.0046 | 0.0049 | -0.688 | -0.797 | 0.3633 | 0.0116 | 0.136 | 0.115 |
| **all auto.** | 1,789 | 1,281,473 | 0.0054 | 0.0036 | 0.006 | 0.0039 | 0.0041 | 0.0045 | -0.715 | -0.7795 | 0.3913 | 0.0111 | 0.149 | 0.114 |
| **X** | 372 | 250,831 | 0.0049 | 0.0014 | 0.0028 | 0.0020 | 0.0049 | 0.0028 | -0.9155 | -1.0725 | 0.6237 | 0.0072 | 0.165 | 0.138 |

**Supp Table 2**. Summary of polymorphism statistics (median) for 4-fold degenerate sites in each chromosome.

| **Frequently-recombining genes** | | | | | | | | | | | | |
| --- | --- | --- | --- | --- | --- | --- | --- | --- | --- | --- | --- | --- |
| **Chrom.** | ***S. lat.* π** | ***S. lat.* π - SD** | ***S. dio.* π** | ***S. dio.* π - SD** | ***S. lat.* adj. π** | ***S. dio.* adj. π** | ***S. lat.* Taj. D** | ***S. dio.* Taj. D** | **F_ST_** | **D_xy_** | ***S. lat.* ZnS** | ***S. dio.* ZnS** |
| **1** | 0.0296 | 0.0176 | 0.0322 | 0.0186 | 0.0222 | 0.0242 | -0.487 | -0.606 | 0.102 | 0.0363 | 0.106 | 0.088 |
| **2** | 0.0316 | 0.0186 | 0.0331 | 0.0194 | 0.0237 | 0.0248 | -0.516 | -0.506 | 0.1165 | 0.0381 | 0.1095 | 0.084 |
| **3** | 0.0338 | 0.0200 | 0.0354 | 0.0217 | 0.0254 | 0.0266 | -0.494 | -0.54 | 0.0935 | 0.0423 | 0.107 | 0.083 |
| **4** | 0.0293 | 0.0169 | 0.0325 | 0.0187 | 0.0220 | 0.0244 | -0.3975 | -0.356 | 0.1397 | 0.0352 | 0.109 | 0.0895 |
| **5** | 0.028 | 0.0172 | 0.0285 | 0.0184 | 0.021 | 0.0214 | -0.49 | -0.37 | 0.0971 | 0.0349 | 0.103 | 0.088 |
| **6** | 0.1206 | 0.0372 | 0.1143 | 0.0351 | 0.0905 | 0.0857 | 2.168 | 2.176 | 0.1014 | 0.1307 | 0.344 | 0.374 |
| **7** | 0.0344 | 0.0192 | 0.036 | 0.0202 | 0.0258 | 0.027 | -0.365 | -0.5205 | 0.0900 | 0.0402 | 0.103 | 0.083 |
| **8** | 0.0321 | 0.0180 | 0.0341 | 0.0200 | 0.0240 | 0.0256 | -0.351 | -0.5575 | 0.0630 | 0.0381 | 0.102 | 0.083 |
| **9** | 0.0319 | 0.0193 | 0.0347 | 0.0199 | 0.0239 | 0.0260 | -0.5855 | -0.503 | 0.0670 | 0.0375 | 0.104 | 0.085 |
| **10** | 0.0317 | 0.0190 | 0.032 | 0.0200 | 0.0238 | 0.024 | -0.6255 | -0.672 | 0.0346 | 0.0337 | 0.097 | 0.082 |
| **11** | 0.0347 | 0.0200 | 0.0343 | 0.0205 | 0.0260 | 0.0257 | -0.459 | -0.421 | 0.0823 | 0.0380 | 0.106 | 0.0825 |
| **all auto.** | 0.0324 | 0.0187 | 0.0341 | 0.0200 | 0.0243 | 0.0256 | -0.471 | -0.52 | 0.0892 | 0.0379 | 0.106 | 0.085 |
| **X** | 0.0227 | 0.0131 | 0.0209 | 0.0129 | 0.0227 | 0.0209 | -0.5 | -0.602 | 0.1713 | 0.0304 | 0.1215 | 0.094 |
| **Rarely-recombining genes** | | | | | | | | | | | | |
| **Chrom.** | ***S. lat.* π** | ***S. lat.* π - SD** | ***S. dio.* π** | ***S. dio.* π - SD** | ***S. lat.* adj. π** | ***S. dio.* adj. π** | ***S. lat.* Taj. D** | ***S. dio.* Taj. D** | **F_ST_** | **D_xy_** | ***S. lat.* ZnS** | ***S. dio.* ZnS** |
| **1** | 0.0108 | 0.0080 | 0.008 | 0.0059 | 0.0081 | 0.006 | -0.734 | -0.543 | 0.3286 | 0.0211 | 0.1645 | 0.098 |
| **2** | 0.0139 | 0.0072 | 0.0141 | 0.0102 | 0.0104 | 0.0105 | -0.3455 | -0.696 | 0.3118 | 0.0262 | 0.126 | 0.117 |
| **3** | 0.0125 | 0.0076 | 0.0169 | 0.0097 | 0.0094 | 0.0126 | -0.5445 | -0.6105 | 0.3011 | 0.0255 | 0.144 | 0.106 |
| **4** | 0.0066 | 0.0046 | 0.0071 | 0.0052 | 0.0050 | 0.0053 | -0.615 | -0.444 | 0.3345 | 0.0157 | 0.1415 | 0.102 |
| **5** | 0.0107 | 0.0066 | 0.0128 | 0.0070 | 0.0080 | 0.0096 | -0.614 | -0.53 | 0.3227 | 0.0215 | 0.124 | 0.092 |
| **6** | 0.012 | 0.0070 | 0.0082 | 0.0065 | 0.0090 | 0.0061 | -0.441 | -0.413 | 0.3259 | 0.0226 | 0.161 | 0.114 |
| **7** | 0.0066 | 0.0050 | 0.0057 | 0.0043 | 0.0050 | 0.0043 | -1.11 | -0.952 | 0.4979 | 0.0157 | 0.175 | 0.102 |
| **8** | 0.0138 | 0.0088 | 0.0163 | 0.0103 | 0.0104 | 0.0123 | -0.5795 | -0.774 | 0.2869 | 0.0247 | 0.142 | 0.111 |
| **9** | 0.012 | 0.0072 | 0.0126 | 0.0080 | 0.009 | 0.0095 | -0.4635 | -0.636 | 0.2837 | 0.0211 | 0.137 | 0.107 |
| **10** | 0.006 | 0.0041 | 0.0084 | 0.0063 | 0.0045 | 0.0063 | -0.984 | -0.842 | 0.4280 | 0.0185 | 0.1255 | 0.1205 |
| **11** | 0.0119 | 0.0075 | 0.0141 | 0.0092 | 0.0089 | 0.0105 | -0.5885 | -0.7075 | 0.2929 | 0.0249 | 0.1235 | 0.1 |
| **all auto.** | 0.0107 | 0.0068 | 0.0118 | 0.0077 | 0.0080 | 0.0089 | -0.608 | -0.638 | 0.3219 | 0.0224 | 0.136 | 0.106 |
| **X** | 0.0029 | 0.0024 | 0.0048 | 0.0039 | 0.0029 | 0.0048 | -0.681 | -1.018 | 0.4725 | 0.0138 | 0.133 | 0.125 |

**Supp Table 3**. Summary of polymorphism statistics (median) for 1^st^ and 2^nd^ codon positions in each chromosome.

| **Frequently-recombining genes** | | | | | | | | | | | | |
| --- | --- | --- | --- | --- | --- | --- | --- | --- | --- | --- | --- | --- |
| **Chrom.** | ***S. lat.* π** | ***S. lat.* π - SD** | ***S. dio.* π** | ***S. dio.* π - SD** | ***S. lat.* adj. π** | ***S. dio.* adj. π** | ***S. lat.* Taj. D** | ***S. dio.* Taj. D** | **F_ST_** | **D_xy_** | ***S. lat.* ZnS** | ***S. dio.* ZnS** |
| **1** | 0.0049 | 0.0031 | 0.0047 | 0.0032 | 0.0037 | 0.0035 | -0.74 | 0.919 | 0.1095 | 0.0058 | 0.1045 | 0.0855 |
| **2** | 0.0049 | 0.0031 | 0.0051 | 0.0034 | 0.0036 | 0.0038 | -0.852 | -0.884 | 0.1070 | 0.0064 | 0.103 | 0.093 |
| **3** | 0.0059 | 0.0038 | 0.0063 | 0.0040 | 0.0044 | 0.0047 | -0.734 | -0.845 | 0.0942 | 0.0072 | 0.1105 | 0.09 |
| **4** | 0.003 | 0.0020 | 0.0037 | 0.0024 | 0.0023 | 0.0027 | -0.7415 | -0.6575 | 0.0905 | 0.0044 | 0.1 | 0.068 |
| **5** | 0.0053 | 0.0033 | 0.0069 | 0.0038 | 0.0039 | 0.0051 | -0.562 | -0.7105 | 0.0510 | 0.0075 | 0.126 | 0.1035 |
| **6** | 0.031 | 0.0138 | 0.0327 | 0.0123 | 0.0233 | 0.0245 | 0.404 | 1.162 | 0.0504 | 0.0336 | 0.172 | 0.282 |
| **7** | 0.0048 | 0.0030 | 0.0045 | 0.0032 | 0.0036 | 0.0033 | -0.6475 | -0.694 | 0.0880 | 0.0060 | 0.108 | 0.091 |
| **8** | 0.0050 | 0.0030 | 0.0053 | 0.0032 | 0.0037 | 0.0040 | -0.662 | -0.734 | 0.0596 | 0.0059 | 0.104 | 0.089 |
| **9** | 0.0055 | 0.0033 | 0.0056 | 0.0038 | 0.0041 | 0.0042 | -0.804 | -0.8305 | 0.0683 | 0.0063 | 0.102 | 0.089 |
| **10** | 0.0061 | 0.0039 | 0.0057 | 0.0045 | 0.0045 | 0.0042 | -0.9 | -1.051 | 0.0278 | 0.0066 | 0.096 | 0.087 |
| **11** | 0.0054 | 0.0036 | 0.0056 | 0.0036 | 0.0041 | 0.0042 | -0.801 | -0.817 | 0.0682 | 0.0065 | 0.114 | 0.089 |
| **all auto.** | 0.0053 | 0.0033 | 0.0053 | 0.0035 | 0.0039 | 0.0040 | -0.7385 | -0.84 | 0.0833 | 0.0064 | 0.107 | 0.089 |
| **X** | 0.0043 | 0.0027 | 0.0039 | 0.0027 | 0.0043 | 0.0039 | -0.692 | -0.733 | 0.1732 | 0.0061 | 0.116 | 0.104 |
| **Rarely-recombining genes** | | | | | | | | | | | | |
| **Chrom.** | ***S. lat.* π** | ***S. lat.* π - SD** | ***S. dio.* π** | ***S. dio.* π - SD** | ***S. lat.* adj. π** | ***S. dio.* adj. π** | ***S. lat.* Taj. D** | ***S. dio.* Taj. D** | **F_ST_** | **D_xy_** | ***S. lat.* ZnS** | ***S. dio.* ZnS** |
| **1** | 0.0026 | 0.0023 | 0.0023 | 0.0018 | 0.0020 | 0.0017 | -1.116 | -0.727 | 0.3988 | 0.0058 | 0.14115 | 0.084 |
| **2** | 0.0027 | 0.0020 | 0.0033 | 0.0025 | 0.0020 | 0.0025 | -0.8 | -0.883 | 0.2813 | 0.0057 | 0.1125 | 0.097 |
| **3** | 0.0025 | 0.0018 | 0.0030 | 0.0023 | 0.0019 | 0.0022 | -0.891 | -0.934 | 0.2674 | 0.0048 | 0.1315 | 0.1045 |
| **4** | 0.0019 | 0.0013 | 0.0018 | 0.0014 | 0.0014 | 0.0014 | -0.921 | -0.802 | 0.3310 | 0.0035 | 0.127 | 0.109 |
| **5** | 0.0025 | 0.0018 | 0.0024 | 0.0017 | 0.0019 | 0.0018 | -0.9625 | -0.8325 | 0.3485 | 0.0046 | 0.118 | 0.0985 |
| **6** | 0.0021 | 0.0017 | 0.0017 | 0.0013 | 0.0016 | 0.0013 | -0.818 | -0.736 | 0.2997 | 0.0037 | 0.162 | 0.108 |
| **7** | 0.0018 | 0.0012 | 0.0019 | 0.0015 | 0.0013 | 0.0014 | -0.993 | -0.972 | 0.3880 | 0.0042 | 0.144 | 0.114 |
| **8** | 0.0030 | 0.0020 | 0.0034 | 0.0024 | 0.0022 | 0.0026 | -0.775 | -0.943 | 0.2775 | 0.0052 | 0.1315 | 0.105 |
| **9** | 0.0024 | 0.0018 | 0.0033 | 0.0021 | 0.0018 | 0.0024 | -0.716 | -0.886 | 0.2555 | 0.0048 | 0.122 | 0.102 |
| **10** | 0.0016 | 0.0013 | 0.0022 | 0.0016 | 0.0012 | 0.0017 | -1.159 | -1.102 | 0.4295 | 0.0044 | 0.136 | 0.1085 |
| **11** | 0.0021 | 0.0017 | 0.0027 | 0.0019 | 0.0016 | 0.0020 | -0.825 | -0.905 | 0.2831 | 0.0041 | 0.111 | 0.098 |
| **all auto.** | 0.0023 | 0.0017 | 0.0026 | 0.0019 | 0.0017 | 0.0020 | -0.8985 | -0.9 | 0.3122 | 0.0045 | 0.128 | 0.103 |
| **X** | 0.0011 | 0.0009 | 0.0016 | 0.0013 | 0.0011 | 0.0016 | -0.887 | -1.092 | 0.5415 | 0.0038 | 0.136 | 0.119 |


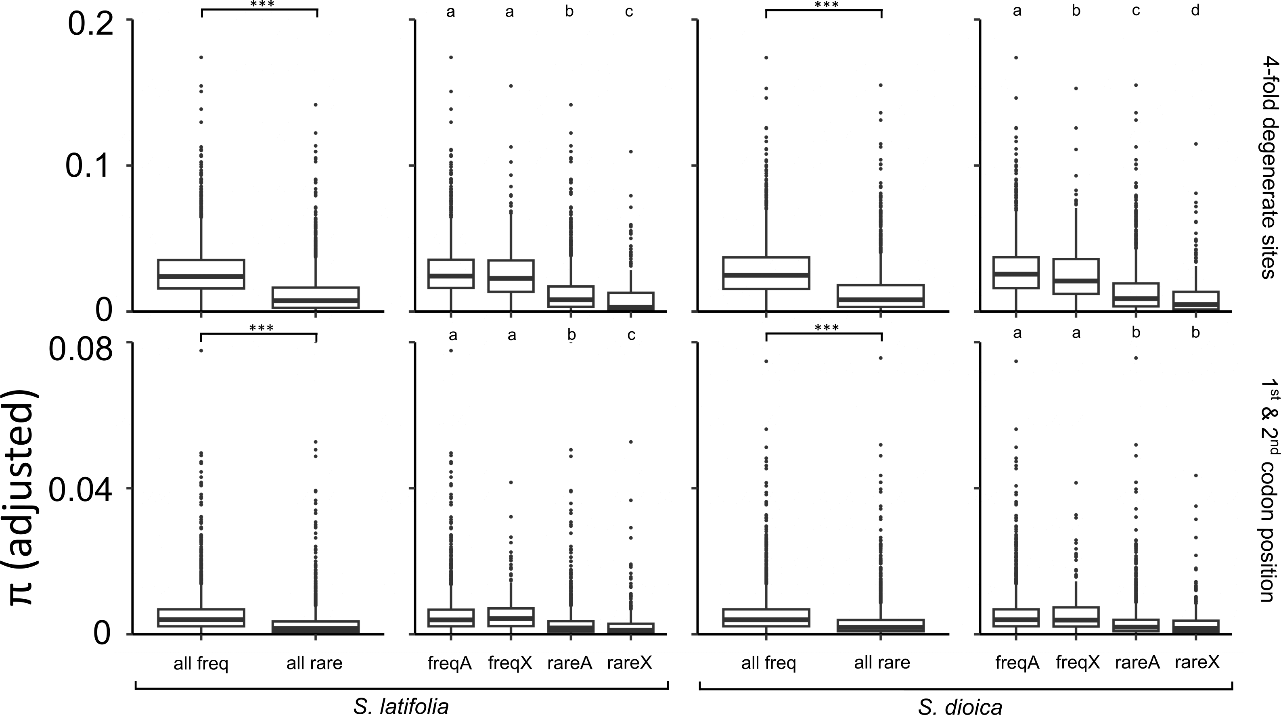


**Supp. Figure 1**. Full figure for adjusted nucleotide diversity (π) in 4-degenerate sites and first two codon positions of different group of genes.


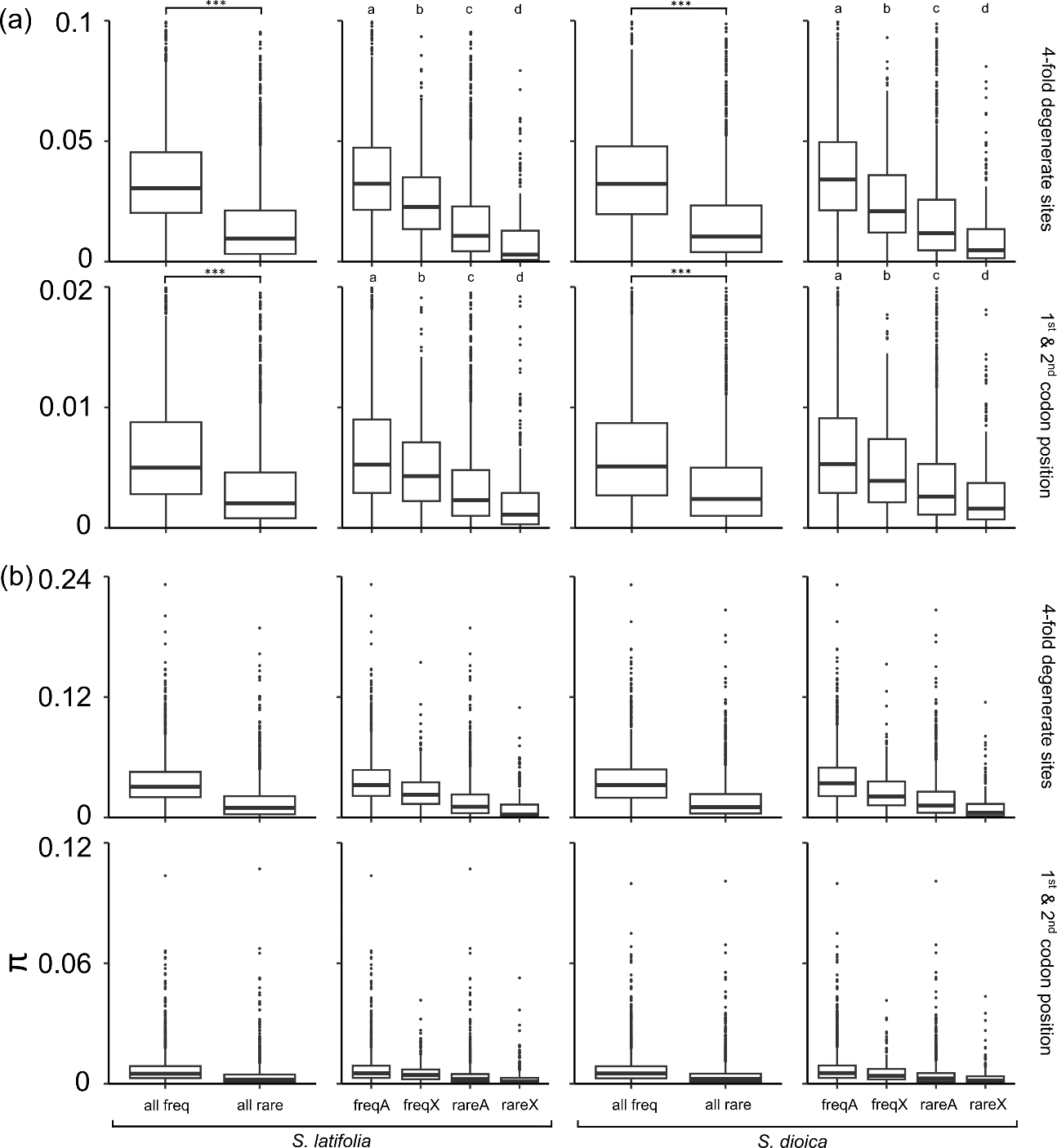


**Supp. Figure 2**. Nucleotide diversity (π) in 4-degenerate sites and first two codon positions of different groups of genes. (a) zoomed-in figures with better resolution of differences among boxes. (b) full figure.


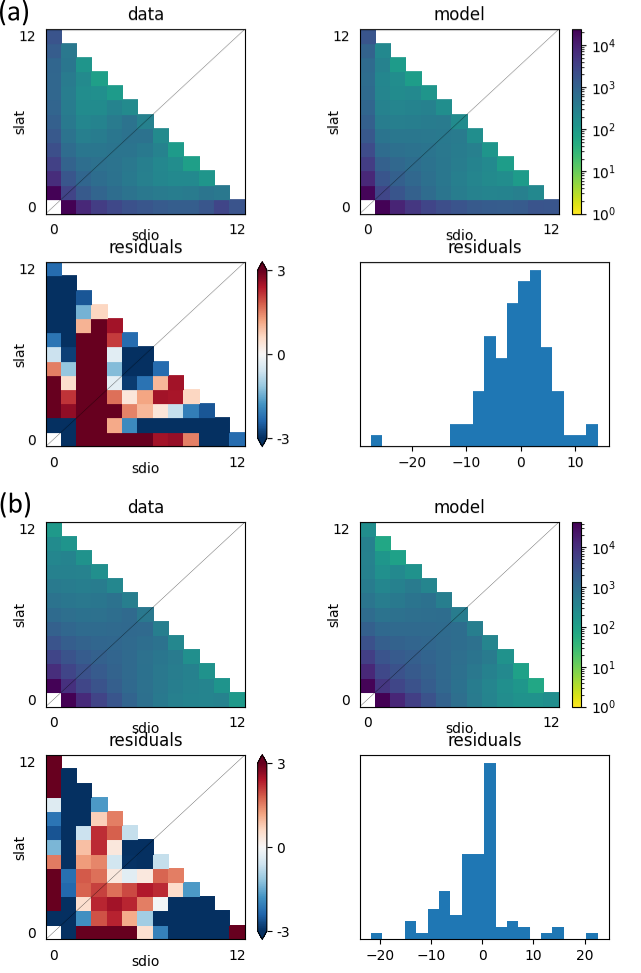


**Supp. Figure 3.** The fit of the best-fitting demographic model (IM2M_2M) to site frequency spectrum data for (a) rarely-recombining genes; and (b) frequently-recombining genes between *S. latifolia* and *S. dioica*. For each gene group (a or b), top left panel = observed two-dimensional site-frequency-spectrum (2D-SFS); top right panel = 2D-SFS expected under the respective model; bottom two panels = residuals between the observed and the expected site-frequency-spectra.
